# Prenatal genetic diagnosis of omphalocele by karyotyping, chromosomal microarray analysis and exome sequencing

**DOI:** 10.1080/07853890.2021.1962966

**Published:** 2021-08-10

**Authors:** Xiaomei Shi, Hui Tang, Jian Lu, Xiue Yang, Hongke Ding, Jing Wu

**Affiliations:** Gentic Medical Center, Guangdong Women and Children Hospital, Guangzhou, PR China

**Keywords:** Omphalocele, prenatal diagnosis, karyotyping, chromosomal microarray analysis, whole-exome sequencing

## Abstract

**Objectives:**

The aim of this study is to share our experience in the prenatal diagnosis of omphalocele by karyotyping, chromosomal microarray analysis (CMA) and whole exome sequencing (WES).

**Methods:**

In this retrospective study, 81 cases of omphalocele were identified from 2015 to 2020. Associated anomalies and prenatal diagnosis based on karyotyping, CMA and WES were analysed.

**Results:**

Fifty-eight (71.6%) of the 81 foetuses had other ultrasound anomalies. Giant omphalocele was present in 11 cases (13.6%) and small omphalocele was present in 70 cases (86.4%). Chromosomal abnormalities were found in 24 foetuses (29.6%, 24/81), the most common of which were trisomy 18 (58.8%, 11/24) and trisomy 13 (29.2%, 7/24). Compared to isolated omphalocele, non-isolated omphalocele was accompanied by an increased prevalence of chromosomal abnormalities (4.3% (1/23) *vs.* 39.7% (23/58), *χ*^2^ = 8.226, *p* = .004). All chromosomal abnormalities were found in small omphalocele. Aside from aneuploidy, CMA showed one pathogenic copy number variants (CNVs) for a detection rate of 1.2%, one variants of unknown significance (VOUS) and one instance of loss of heterozygosity (LOH). WES was performed on 3 non-isolated cases, and one was found to have pathogenic variants.

**Conclusions:**

The most common genetic cause of omphalocele is aneuploidy and the prevalence of chromosomal abnormalities is increased with non-isolated and small omphalocele. CMA and WES can be useful for providing further genetic information to assist in prenatal counselling and pregnancy management.

## Introduction

Omphalocele is a congenital defect in the abdominal wall characterized by absent abdominal muscles, fascia and skin and is one of the most common types of abdominal wall defects. Omphalocele is easily recognizable on ultrasound examination. Approximately, 50% of cases are associated with multiple malformation syndromes, including cardiac, gastrointestinal, genitourinary, musculoskeletal and central nervous system abnormalities [1]. It has been reported that chromosomal abnormalities are present in 30–70% of cases where omphalocele is accompanied by other malformation syndromes. The most frequent chromosomal anomalies are trisomies 18 and 13, pentalogy of Cantrell and Beckwith–Wiedemann syndrome (BWS), and autosomal dominant and X-linked inheritance [2,3]. Therefore, understanding the genetic abnormalities underlying omphalocele is important for the diagnosis of this disease, which will aid in parental counselling and assist pregnancy management.

G-banding karyotyping has been the predominant strategy applied for detecting chromosomal abnormalities in foetuses with omphalocele in clinical practice over the past few decades. However, it has some limited resolutions. It is time-consuming and low-resolution. Chromosomal microarray analysis (CMA) has been recommended as the first-tier test for foetal structural abnormalities in identification of microscopic or submicroscopic copy number variants (CNVs). However, CMA cannot detect single-nucleotide variants (SNVs) and small insertions/deletions. With advances in genetic technology, whole-exome sequencing (WES) has been recently introduced. According to the PAGE cohort study, the yield of WES was 8.5% in foetuses with structural defects after the exclusion of aneuploidies and CNVs [4]. However, there are very few case reports of prenatal diagnosis of omphalocele detected by CMA or WES in the literature. We designed a retrospective study of foetuses with omphalocele undergoing karyotyping, CMA and WES at our institution over five years to evaluate their feasibility in prenatal diagnosis for foetuses with omphalocele.

## Materials and methods

This retrospective study was conducted at Guangdong women and children hospital between 2015 and 2020. The study was approved by our institutional review board and clinical research ethics committee. Foetuses were included if they had a prenatal ultrasound report showing omphalocele.

Giant omphalocele was defined by abdominal wall defects larger than 5 cm in diameter. Small omphalocele was defined by abdominal wall defects smaller than 5 cm in diameter. Isolated omphalocele was defined as sole omphalocele. Non-isolated omphalocele was defined as omphalocele in combination with another soft marker or a structural abnormality.

After diagnosis, all parents were given comprehensive counselling. Karyotyping and CMA analysis were presented to all foetuses. When no abnormalities were detected by karyotyping and CMA, WES was offered as an option.

Metaphase chromosome G-banding karyotyping was performed at a level of 320–400 bands. CMA was performed using a high-resolution genotyping single nucleotide polymorphism microarray, Affymetrix CytoScan 750 K Array (Affymetrix, Santa Clara, CA). CNVs were identified based on records associated with the human reference genome 37(NCBI37hg19) of the National Centre for Biotechnology Information.

Subsequently, genomic DNA samples from parent–foetus trios were subjected to WES using an Illumina Nextera Rapid Capture Exome Kit for library preparation. Sequencing was performed with the HiSeq 2500 platform (Illumina, San Diego, CA). Only variants related to the phenotype were reported. Variants were classified according to the American College of Medical Genetics and Genomics (ACMG) guidelines by the laboratory.

Statistical analysis was performed with the SPSS version 24.0 (IBM, Armonk, NY). Quantitative variables are expressed as the mean ± standard deviation, and categorical variables are expressed as the frequency and percentage. Differences between categorical variables were analysed using Chi-square test or Fisher’s exact test. *p* < .05 was considered statistically significant.

## Results

There were 81 foetuses prenatally diagnosed with omphalocele during the study period. The median maternal age at the time of sampling was 30.9 ± 5.4 years and the gestational age at the time of invasive prenatal diagnosis was 18.0 ± 5.7 weeks. The methods of invasive test were chorionic villus sampling in 31 cases (38.3%, 31/81), amniocentesis sampling in 41 cases (50.6%, 41/81) and cordocentesis sampling in 9 cases (11.1%, 9/81).

Among these foetuses, there were 23 cases (28.4%, 23/81) of isolated omphalocele and 58 cases (71.6%, 58/81) with other ultrasound anomalies. There were 11 cases of giant omphalocele (13.6%, 11/81) and 70 cases of small omphalocele (86.4%, 70/81).

### Prenatal diagnostic results of karyotyping

Karyotyping results showed that chromosomal abnormalities were found in 24 foetuses, including 23 aneuploidies and 1 unbalanced translocation (Table 1). Overall, the prevalence of chromosomal abnormalities was 29.6% (24/81). The most common chromosomal abnormalities were trisomy 18 (58.8%, 11/24) and trisomy 13 (29.2%, 7/24), followed by trisomy 21(16.6%, 4/24), Klinefelter’s syndrome (4.2%, 1/24) and unbalanced translocation (4.2%, 1/24).

**Table 1.** Abnormal karyotype in foetuses with omphalocele.

NO	MA	GA	Size of omphalocele	Other ultrasound findings	Karyotype results	Pregnant outcome
1	38	12	Small	Nuchal translucency thickening	Trisomy 18	TOP
2	30	13	Small	Hydroderma and nuchal translucency thickening	Trisomy 18	TOP
3	25	14	Small	Nuchal translucency thickening	Trisomy 18	TOP
4	40	21	Small	Double outlet right ventricle and ventricular septal defects	Trisomy 18	TOP
5	41	20	Small	Choroid plexus cysts	Trisomy 18	TOP
6	39	13	Small	Hydroderma and cystic hygroma	Trisomy 18	TOP
7	40	19	Small	Single umbilical artery, ventricular septal defects and Aortic riding	Trisomy 18	TOP
8	39	13	Small	Nuchal translucency thickening and single umbilical artery	Trisomy 18	TOP
9	30	14	Small	Nuchal translucency thickening, hydroderma and cystic hygroma	Trisomy 18	TOP
10	31	13	Small	Nuchal translucency thickening, hydroderma and cystic hygroma	Trisomy 18	TOP
11	35	23	Small	Echogenic bowel, ventricular septal defects and overlapping fingers	Trisomy 18	TOP
12	34	16	Small	Nuchal translucency thickening, holoprosencephaly, cleft lip and palate	Trisomy 13	TOP
13	25	13	Small	Nuchal translucency thickening and cystic hygroma	Trisomy 13	TOP
14	30	17	Small	Polydactyly	Trisomy 13	TOP
15	27	12	Small	Nuchal translucency thickening and cardiac defects	Trisomy 13	TOP
16	38	14	Small	Nuchal translucency thickening, absent nasal bone and umbilical cord cyst	Trisomy 13	TOP
17	37	24	Small	Hydronephrosis, cardiac defects, nuchal fold thickening and echogenic bowel	Trisomy 13	TOP
18	25	13	Small	Nuchal translucency thickening, cystic hygroma and cardiac defects	Trisomy 13	TOP
18	31	18	Small	echogenic bowel	Trisomy 21	TOP
20	35	13	Small	Nuchal translucency thickening	Trisomy 21	TOP
21	35	11	Small	Nuchal translucency thickening and cystic hygroma	Trisomy 21	TOP
22	23	13	Small	/	Trisomy 21	TOP
23	29	16	Small	Nuchal translucency thickening, cardiac defects and deformity of lower extremity	47,XXY	TOP
24	25	12	Small	Cystic hygroma, holoprosencephaly, cleft lip and palate and hydrops	46,XN,rec(7)dup(7p)inv(7)(p21q35)	TOP

MA: Mean maternal age; GA: gestational age.

**Figure 1.**
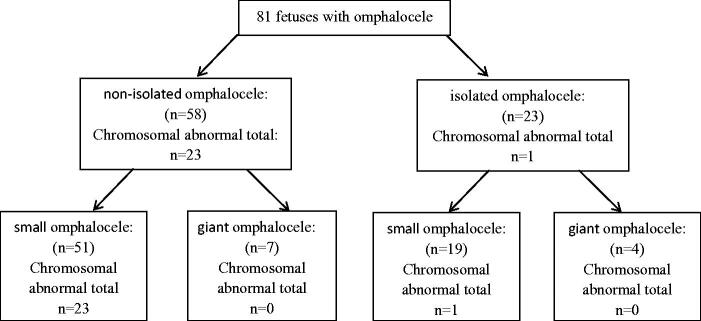
Prenatal diagnosis for isolated and non-isolated omphalocele.

In isolated omphalocele, chromosomal abnormalities were found in 1 foetus (trisomy 21) at 12 weeks. In non-isolated omphalocele, chromosomal abnormalities were found in 23 foetuses. Compared to isolated omphalocele, the prevalence of chromosomal abnormalities in non-isolated omphalocele was increased (4.3 *vs.* 39.7%, *χ*^2^=8.226, *p* = .004, Chi-square test for continuous calibration). In giant omphalocele, no chromosomal abnormalities were found, and all were found in small omphalocele (Figure 1).

### Prenatal diagnostic results of CMA

Of the 81 cases in which CMA was performed, aneuploidy was detected in 23 cases. In addition, there was 1 case of pathogenic CNVs, 1 case of VOUS and 1 case of LOH. CMA results (except aneuploidy) are outlined in Table 2. The total yield of pathogenic CNVs in CMA testing was 1.2% (1/81).

**Table 2.** Abnormal CMA (except aneuploidy) in foetuses with omphalocele.

NO	MA	GA	Size of omphalocele	Other ultrasound findings	Karyotype results	CMA results	Further analysis and outcomes
1	25	12	Small	Cystic hygroma, hydrops holoprosencephaly, cleft lip and palate	46,XN,rec(7)dup(7p) inv(7)(p21q35)	7p22.3p21.3(162,702-7,332,921)x3 7q35q36.3(143,218,383-159, 119,707)x1	G banding of father： 46,XY, inv(7)(p21q35) TOP
2	29	13	Small	Placentomegaly	Normal	11p15.5(2,344,807-2,520,511)x1 (VOUS)	MS-MLPA and confirmed BWS TOP
3	35	11	Small	Cystic hygroma and nuchal translucency thickening	T21	(14)×2 hmz,(21)×3	No detect, TOP since T21

MA: Mean maternal age; GA: gestational age; TOP: termination of pregnancy.

In Case 2, omphalocele and placentomegaly were prenatally diagnosed by ultrasound. CMA showed a microdeletion in 11p15.5, which may cause DNA methylation aberrance leading to Beckwith–Wiedemann syndrome. Methylation-sensitive multiplex ligation probe analysis (MS-MLPA) revealed a loss of methylation at imprinting control region 2 and a 50% reduction of copy numbers of *KCNQ1* gene. We finally confirmed the diagnosis of Beckwith–Wiedemann syndrome [5].

In Case 3, the foetus was diagnosed with omphalocele, cystic hygroma and increased nuchal translucency and had already been diagnosed with trisomy 21 based on the prenatal karyotype. LOH at chromosome 14 suggests the existence of uniparental disomy (UPD) (14) and may be associated with Temple syndrome or Kagami–Ogata syndrome. Unfortunately, further analysis could not be performed because the pregnancy was terminated.

### Prenatal diagnostic results of WES

**Figure 2.**
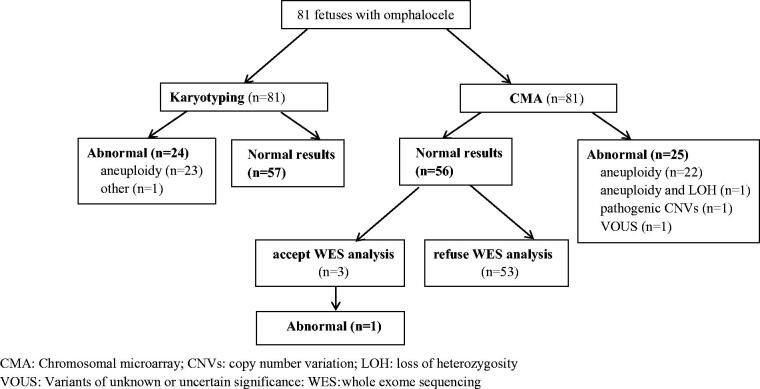
Prenatal diagnosis for foetuses with omphalocele according to the type of genetic analysis. CMA: chromosomal microarray; CNVs: copy number variation; LOH: loss of heterozygosity; VOUS: Variants of unknown or uncertain significance: WES: whole-exome sequencing.

After the exclusion of aneuploidies and pathogenic CNVs, there were 56 cases. Three non-isolated omphalocele cases accepted WES, and one had pathogenic variants (Figure 2).

**Figure 3.**
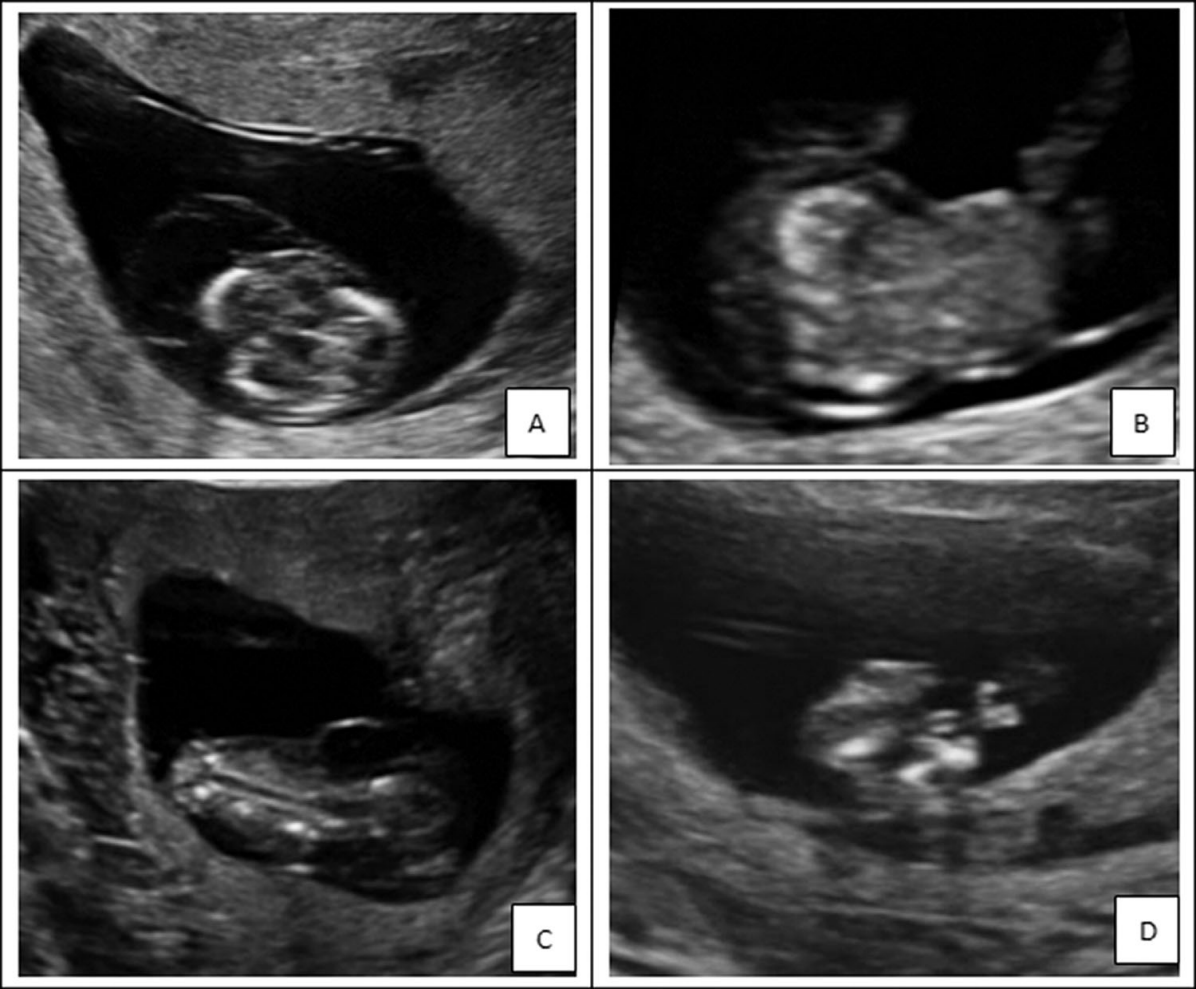
The ultrasound examination of the case with diastrophic dysplasia. The ultrasound examination showed that cystic hygroma (A), omphalocele (B), spinal malformation (C) and short limbs (D).

In this case, omphalocele, cystic hygroma, spinal malformation and short limbs were prenatally diagnosed by ultrasound at 11^+3 ^weeks (Figure 3). Moreover, the woman had two similar pregnancies before. WES showed a heterozygous point variation of the *SLC26A2* gene: c.1020_1022delTGT (p.Val341del) from the father and the heterozygosity exon 2–3 deletion from the mother, forming a compound heterozygosity. Mutations in *SLC26A2* gene result in a spectrum of autosomal recessive chondrodysplasias that range from the mildest recessive form of multiple epiphysial dysplasia (rMED) through the most common diastrophic dysplasia (DTD) to lethal atelosteogenesis type II and achondrogenesis IB.

DTD (OMIM 222600) is a rare osteochondrodysplasia characterized by short-limbed short stature and joint dysplasia. DTD is caused by mutations in SLC26A2. The variant c.1020_1022delTGT (p.Val341del) has been previously reported and classified as pathogenic. The deletion of exon 2–3 has not been reported, but was predicted to be a disease-causing variant by *in-silico* prediction tools and classified as likely-pathogenic. Finally, the foetus was diagnosed with DTD by combined prenatal ultrasonography and whole exome sequencing (WES).

## Discussion

In the literature, Omphalocele is complicated with other organ anomalies and chromosomal abnormalities in 30–70% of cases. The most frequent chromosomal anomalies of omphalocele are trisomies 18 and 13, and small omphaloceles are more likely to be associated with abnormal karyotypes [6–8]. In our study, karyotyping showed that the prevalence of chromosomal abnormalities was 29.6%, and trisomy 18 and trisomy 13 accounted for 58.8 and 29.2%, respectively. Our study also demonstrated that 71.6% of cases had other ultrasound anomalies. For isolated cases, the prevalence of chromosomal abnormalities was 4.3%, while 39.7% in non-isolated omphalocele cases. Moreover, all chromosomal abnormalities were found in small omphalocele. These results were consistent with previous research and further support the idea that the prevalence of chromosomal abnormalities was increased with non-isolated and small omphalocele.

Khalil et al. [9] recommend delaying foetal karyotyping until after the early second-trimester scan in cases of omphalocele with normal nuchal translucency. In their cohort, they observed that many of the omphalocele cases had resolved by 16 weeks of gestation. However, as reported in our study, one foetus with isolated omphalocele was diagnosed with trisomy 21 at 12 weeks. Therefore, isolated omphalocele may be associated with chromosomal abnormalities even in the first-trimester, so we suggest that prenatal diagnosis should be presented to all foetuses with omphalocele.

It has been shown that CMA has the ability to detect clinically significant CNVs in 6–8% of pregnancies with ultrasound abnormalities [10]. However, previous studies looking at prenatal CMA do not specifically report cases of omphalocele, therefore, the exact utility of CMA in cases of omphalocele remains unclear. In our study, there was 1 case (1.2%) of pathogenic CNVs, and the most likely explanation for the low yield of CMA in cases of omphalocele is that most common genetic cause of omphalocele is due to aneuploidy, rather than CNVs. Jasmin Beygo et al. [11] reported that a maternal deletion upstream of the imprint control region 2 in 11p15 causes loss of methylation and familial Beckwith–Wiedemann syndrome. In our study, CMA found an 11p15.5 microdeletion in a foetus affected by omphalocele and placentomegaly. As the deletion also includes the promoter and 5′ part of the KCNQ1 gene, we considered it may cause BWS. To verify the initial clinical diagnosis of BWS, molecular genetic analysis was performed using MS-MLPA of chromosome 11p15 and finally confirmed the diagnosis [5]. We also found a LOH at chromosome 14 in a foetus. UPD (14) may be associated with Temple syndrome or Kagami–Ogata syndrome. Temple syndrome and Kagami–Ogata syndrome are both rare imprinting disorders. These results demonstrate that CMA is a valuable tool for identifying unbalanced submicroscopic chromosomal abnormalities and LOH in the prenatal diagnosis of omphalocele.

BWS is the most common paediatric overgrowth syndrome, classically characterized by omphalocele, macroglossia and overgrowth. Omphalocele can be associated with BWS. Omphaloceles have seen with BWS often contain only the small bowel, and additional findings in BWS include macrosomia, organomegaly, macroglossia, polyhydramnios, and placentomegaly [12,13]. Previous studies have reported BWS in association with prenatally diagnosed omphalocele in the range of 2–23% [14,15]. Abbasi N et al. [16] conducted a retrospective review of 92 prenatally diagnosed omphalocele with molecular confirmation of BWS and showed that BWS was identified in 37 and 7% of isolated and non-isolated omphalocele, respectively, after exclusion of aneuploidy. However, we found only one case (1.2%, 1/81) of BWS in our study. Multiple possible explanations exist, and a potential reason for this discrepancy is that major underlying pathogenetic mechanisms of BWS include loss or gain of DNA methylation, paternal UPD, chromosomal rearrangements and gene sequencing. However, in our study, we did not provide MS-MLPA to all foetuses, which may lead to missed diagnosis in some cases. Another possible reason is that many of the features of BWS develop late in gestation or postnatally, and some foetuses with non-isolated omphalocele were terminated prior to expanded molecular analysis.

A wide spectrum of genetic disorders has been reported in association with omphalocele. These disorders, such as Donnai–Barrow syndrome, acrocallosal syndrome and hydrolethalus syndrome, are not detected by either CMA or karyotype and are likely to explain a significant portion of unexplained cases. It may be reasonable to consider exome sequencing as the next step when standard genetic tests do not yield a diagnosis. A recent report shows the prenatal use of WES in diagnosing Donnai–Barrow syndrome in the setting of omphalocele and associated anomalies [17]. In our study, WES was performed to identify possible causal variants in three non-isolated omphalocele cases, and one pathogenic variant was successfully identified. Thus, WES should be a part of the diagnostic workup for any euploid foetus with non-isolated omphalocele, particularly if abnormal pregnancies have occurred repeatedly, as in our case.

## Conclusion

In summary, our study demonstrates that the most common genetic cause of omphalocele is due to aneuploidy, and the prevalence of chromosomal abnormalities was increased with non-isolated and small omphalocele. CMA and WES can be useful in providing further genetic information to assist in prenatal counselling and pregnancy management. This study was limited by its small sample size and retrospective design taking place at a single subspecialty referral centre. Another limitation is that genetic testing, such as MS-MLPA for BWS is not offered for all foetuses. Further investigation through multicentre and prospective research is warranted to evaluate the prenatal diagnosis of omphalocele.

## Data Availability

All data included in this study are available upon request by contact with the corresponding author.
